# Massive corals maintain a positive carbonate budget of a Maldivian upper reef platform despite major bleaching event

**DOI:** 10.1038/s41598-019-42985-2

**Published:** 2019-04-24

**Authors:** E. J. Ryan, K. Hanmer, P. S. Kench

**Affiliations:** 1 School of Environment, the University of Auckland, Auckland, 1010 New Zealand; 2 Department of Earth Sciences, Simon Fraser University, Burnaby, BC Canada

**Keywords:** Tropical ecology, Geomorphology

## Abstract

Coral reefs experienced the third global bleaching event in 2015–2016 due to high sea-surface temperature (SST) anomalies. Declines in net carbonate production associated with coral bleaching are implicated in reef structural collapse and cascading impacts for adjacent coral reef islands. We present the first carbonate budget study of a reef platform surface (reef crest and reef flat) in the southern Maldives and the first record of upper reef flat condition in the central Indian Ocean post the 2015–2016 coral bleaching event. Scleractinian corals were the primary carbonate producers, with live coral cover averaging between 11.1 ± 6.5 and 31.2 ± 21.8% and dominated by massive corals. Gross carbonate production rates averaged 5.9 ± 2.5 G (kg CaCO_3_ m^2^ yr^−1^). Bioerosion was estimated at 3.4 ± 0.4 G, resulting in an average net carbonate production rate of 2.5 ± 2.4 G. Comparison of results with a study of the fore-reef slope highlights major differences in post-bleaching carbonate budget state between the fore-reef slope and the reef platform surface. The positive reef flat carbonate budget is attributed to the persistence of massive corals (*Porites* spp. and *Heliopora* spp.) through the bleaching event.

## Introduction

Tropical coral reefs provide important biological functions and ecosystem goods and services, including dissipation of ocean wave energy, food and building resources, and income through tourism^1–3^. The ability of reefs to maintain these functions in the future is uncertain, as coral reefs globally face multiple natural stressors, such as tropical cyclones/hurricanes^4^ and changes in sea-surface temperature (SST)^5^, which threaten the health and building capacity of reef systems. These natural stressors are compounded by anthropogenic factors including global climate change and pollution^6,7^. Indeed, periods of elevated SST just 1–2 °C above average summer SSTs can lead to coral bleaching, whereby temperature stressed scleractinian (reef-building) corals expel their symbiotic algae (zooxanthellae) from their coral tissue^8^, leaving the corals with a bleached appearance, with only the aragonite skeleton visible through translucent tissue. Bleaching may lead to coral mortality and bleaching/mortality can occasionally be widespread globally, often referred to as a mass global bleaching event^9,10^. Reefs affected by coral bleaching can experience reduced live coral cover^5^, reduced reef rugosity and consequent decreases in reef biodiversity^11^, carbonate production and reef accretion^12^. The third recorded mass global bleaching event occurred in 2015–2016 when El Niño Southern Oscillation (ENSO)-induced warm SST anomalies caused extensive coral bleaching and mortality in tropical waters throughout the Indo-Pacific and the western Atlantic Ocean^10,13,14^. The 2015–2016 coral bleaching event has been associated with declines in reef ecosystem functionality, reef productivity and reef carbonate budgets^15,16^. In the southern Republic of Maldives, SST persisted above the regional bleaching threshold of ~30.9 degrees Celsius for around three months from late March to mid-May 2016^16^, leading to significant declines in live coral cover on fore-reef slopes. Observed reduction in live coral cover resulted in a collapse in reef carbonate budget state, with calcium carbonate production on these Maldivian fore-reefs switching from net positive (pre-bleaching) to net negative (post-bleaching), ultimately resulting in an inferred negative reef accretionary status^16^.

Carbonate budgets are one measure of reef condition that reflects the net amount of calcium carbonate produced within a reef system, and balances the biological accretion and erosion processes operating on an individual reef^17–19^. The primary carbonate producers on coral reefs are corals, with calcifying algae (crustose coralline algae and *Halimeda* spp.) contributing to secondary carbonate production, alongside other calcifying organisms (such as foraminifera and molluscs) and abiotic/microbial cementation. Organisms contributing to bioerosion include endolithic micro- and macro-boring organisms, parrotfish and echinoderms. Temporal shifts in carbonate budget state can be used as an indicator of trends in reef health and provide extended insights into biological erosion^20^, reef framework production and reef accretion potential^12,21,22^, compared with standard ecological abundance surveys, which do not consider the effects of net carbonate production or removal^23–25^.

Understanding the effects of mass coral bleaching and mortality on reef carbonate budgets can also provide useful insights into a reef’s accretion potential under current and future sea-level rise scenarios^12^. The temporal dynamics of carbonate budgets can have broader implications for reef systems and adjacent landforms. For example, recent collapses in reef carbonate budgets observed in the southern Maldives following the 2016 bleaching event^16^ could have cascading impacts for the geomorphic maintenance and stability of adjacent low-lying reef sedimentary landforms (beaches and islands). In the short-term (seasons to years), impacts may include reef or island accretion due to elevated carbonate sediment production associated with increased physical and biological erosion of dead coral. Sediment produced may be supplied to islands or contained within the reef framework and cemented, thus contributing to accretion. However, additional sediment production may also be transported off the reef system. In the longer-term (decades to centuries) reef island stability may be compromised due to a reduction in coral growth and the reef’s capacity to provide a physical barrier to incident wave processes that modulate beach and island erosion. Impacts may be compounded under future scenarios of sea-level rise, promoting widespread erosion and frequent island overtopping^12,26,27^. Such outcomes are of global concern, given recent suggestions that many coral reefs will be unable to continue accreting vertically at a sufficient pace to keep up with rates of projected global sea-level rise^12^. However, there are multiple interacting factors that will contribute to future reef accretion rates, which remain poorly resolved. These factors include: hydrodynamics and the rate of sea-level rise; the contribution of reef flat carbonate production to overall reef accretion; sediment delivery, retention and removal^28,29^ (including storm deposits); biological reef-binding processes (crustose coralline algae); microbial cementation; algal rim accretion rates; the role of carbonate production on sand flats, patch reefs and seagrass meadows; and biochemical dissolution of carbonate framework and sediment^30^.

Despite their recognized importance, carbonate budgets have been estimated for few reefs globally, most of which are located in the Caribbean^17–19,22,31,32^. However, recent work in the past couple of decades has expanded the growing knowledge of reef carbonate budgets to the central Indian Ocean^13,16,33–35^ and elsewhere in the Indo-Pacific^22,29,36,37^. A recent global analysis of carbonate budget data^12^ indicates that net carbonate budgets of reefs in the Indo-Pacific and tropical western Atlantic are currently low and are sub-optimal for reef accretion potential. Despite the growing number of studies of reef carbonate budgets, the overwhelming majority of data are derived from fore-reef slopes between 2–10 m depth^13,16,18,31,34,38^, where conditions for data collection are optimal (i.e. high visibility, low wave energy). The contribution of the reef crest and reef platform surface (upper surface at ~0–1 m depth) carbonate producers to the overall reef budget is often not quantified, with few studies reporting reef flat carbonate budget rates^22,36,37^. This disparity in focus of studies is at odds with the fact that the reef crest and upper reef flat are critical environments when relating reef carbonate budgets to reef accretionary potential under sea-level rise and associated wave impacts at adjacent shorelines. The critical nature of these geomorphic sub-components rests on the importance of the reef crest and reef flat as the key wave energy dissipation zone on a reef^1,39^. Studies have shown that 94–98% of incident incoming wave energy is dissipated as waves interact with the reef edge and propagate across the reef flat^1^. Consequently, it is the residual energy from these interactions that controls the available energy to stimulate sediment deposition/removal at island shorelines^40,41^. Although the fore-reef slope environments play a key functional role in coral reef ecosystems, the potential for vertical reef accretion and the associated impact of theoretically higher water levels on reef flats under sea-level rise scenarios is largely dependent on carbonate production (coral growth and sediment production) and removal processes (including sediment losses, bioerosion and coral mortality) operating at the reef crest and upper reef flat. Furthermore, the reef crest and reef flat are critical components of the carbonate factory for sediment supply to reef islands^42,43^, while sediment produced on the fore-reef slope is largely exported from the reef sytem^44,45^.

**Figure 1:**
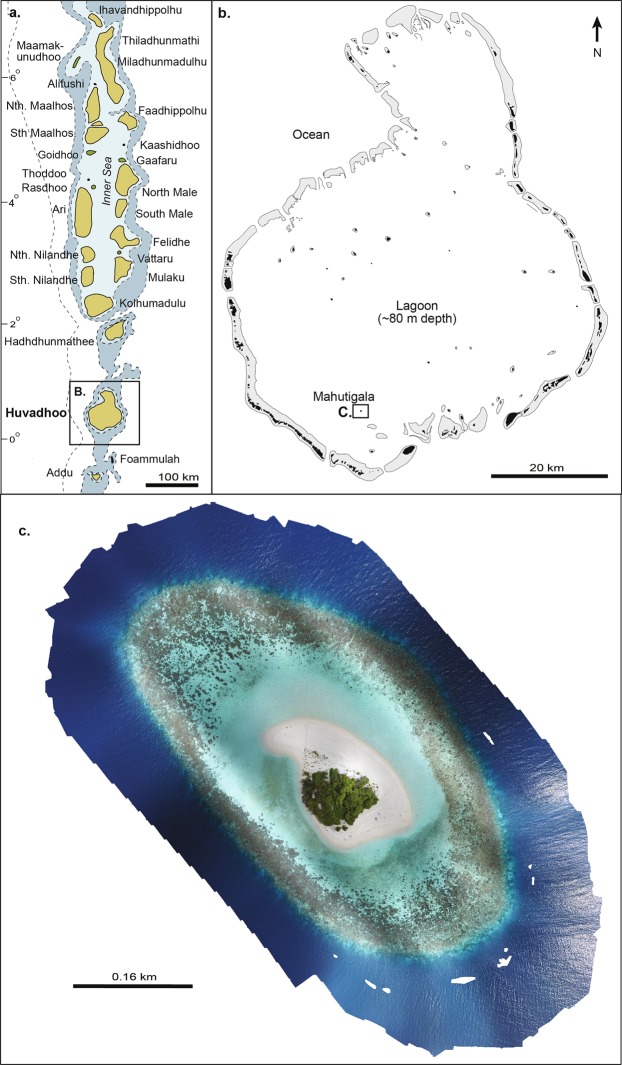
(**a**) Location of Huvadhoo Atoll in the Republic of Maldives, (**b**) Location of Mahutigala reef within the lagoon of Huvadhoo Atoll, (**c**) Mahutigala reef platform (drone imagery taken in February 2017).

Here we present the first ecological survey of a coral reef flat in the Indian Ocean following the 2015–2016 global mass bleaching event. We quantify the carbonate budget of Mahutigala reef platform, a low-energy, sub-tidal, lagoonal platform reef located in Huvadhoo Atoll, Maldives (0°17′22.77′′N, 73°12′1.88′′E, Fig. 1). Significantly, we present the gross carbonate production (by corals and coralline algae) and biological erosion to quantify the net carbonate production of the reef platform surface (G, where G = kg CaCO_3_ m^2^ yr^−1^). We examine reef flat-wide net biological carbonate production with high spatial coverage of observations and evaluate the effect of the 2016 warm SST anomaly event on the reef flat carbonate budget. We highlight the differential roles of coral genera and growth morphologies in the carbonate budget of the reef platform and discuss the important contribution of bleaching-resilient massive corals (such as *Porites* spp.) to the reef flat live coral assemblage following major stress events.

## Results

### Carbonate production

**Figure 2:**
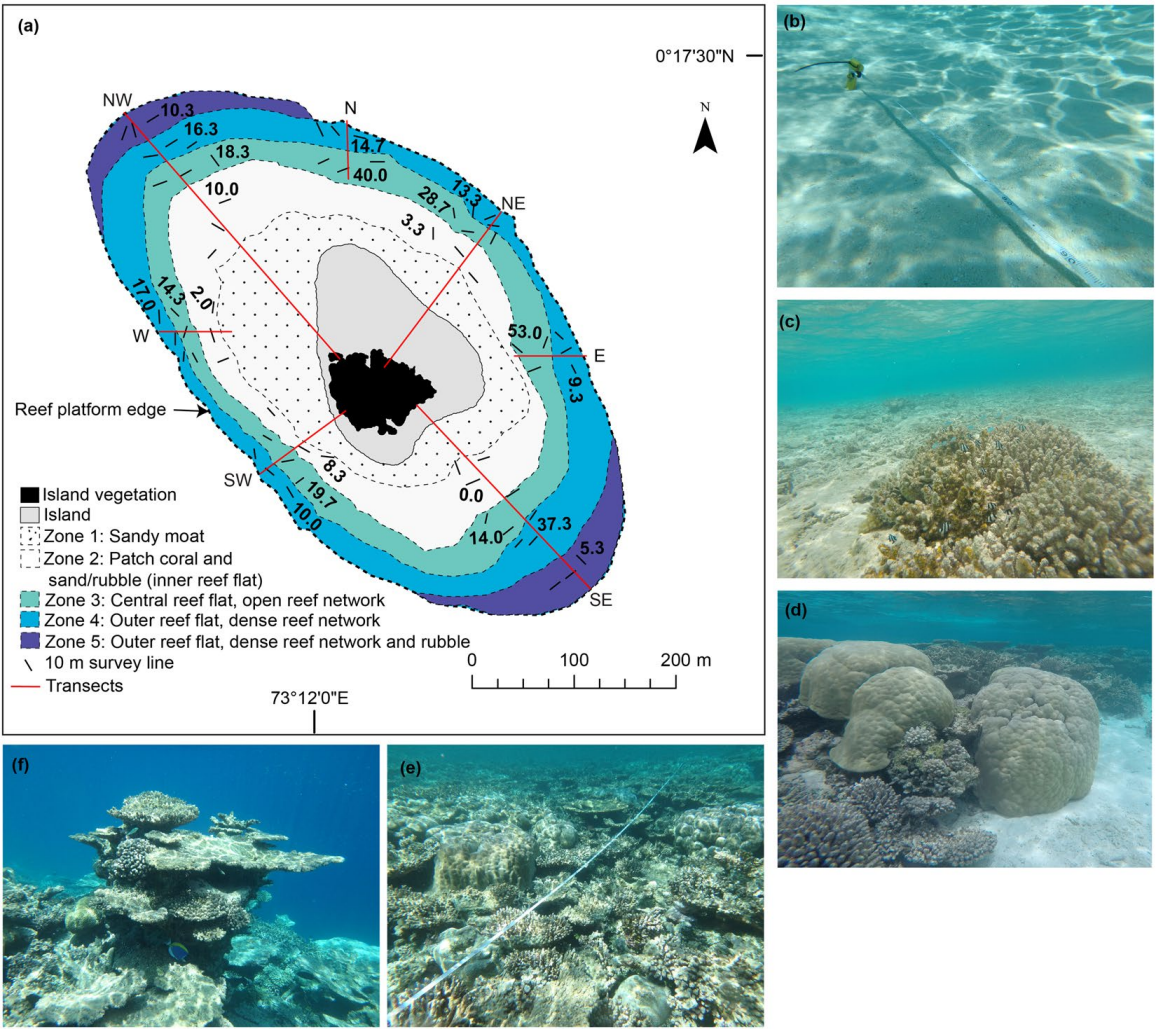
(**a**) Extent of eco-geomorphological zones with island and island vegetation mapped using a Trimble GPS. Zones 1–5 are estimated based on field surveys and extrapolated across the platform using drone imagery. Transects are shown in red and are labelled. Average live coral cover (%) is shown by the numbers in each zone for each transect. Representative photographs of the sandy moat/zone 1 (**b**), the patch reef zone/zone 2 (**c**), the central reef flat/zone 3 (**d**), the outer reef flat/zone 4 (**e**) and the outer reef flat/zone 5 (**f**) are shown.

Five eco-geomorphological zones were differentiated on the Mahutigala reef surface based on 6,300 observations along seven radial transects around the reef platform. Zones were identified based on variations in substrate type including: sand and rubble cover, live and dead coral cover and coral morphology (Fig. 2). An inner sandy moat (zone 1) surrounding the vegetated island (extending 20–100 m seaward of the beach toe) is lower in elevation than outer reef zones (0.8–1.0 m below mean sea level (MSL)) and is characterized by a rippled medium-size sand. Occupying 20% of the platform surface, this sand zone contained no primary or major secondary carbonate producers (i.e. corals, CCA and calcifying macroalgae). Thus, the sandy moat (zone 1) is not considered in ecological analyses hereafter. A patch reef zone (zone 2) extending 20–90 m seaward of the sandy moat was characterized by isolated coral patches (comprising dead and living coral) that stand up to 1.5 m above a mixed sand and coral rubble substrate (occupying ~60–80% of zone 2). Representing the transition from the sandy moat to the inner reef flat, the patch reef zone grades into a narrow central reef flat (zone 3, 10–30 m wide), which is higher in elevation (~0.56 m below MSL). Zone 3 was characterized by an open framework of dead coral (~20–40% cover), dominated by branching and digitate growth morphologies, and live coral (~15–20% cover), predominantly of massive growth morphology, with remaining substrate comprising sand. The outer reef flat (comprising zones 4 and 5) extended a further 20–80 m seaward of the inner reef flat to the reef crest, at a slightly lower elevation (~0.6–0.9 m below MSL). These zones were characterized by a denser network of reef framework comprising dead coral (~30–50% cover) of branching, digitate and tabular morphologies, and living massive coral (~10–30% cover). Dead coral colonies were often covered in filamentous turf algae and/or crustose coralline algae, and this was characteristic of all eco-geomorphic zones where dead coral was recorded. Furthermore, macroalgae (e.g. *Halimeda* spp. and *Peyssonnelia* sp.) colonized areas of available substrate in the inner and outer reef zones. *Peyssonnelia* sp. was often found colonizing the available dead substrate and crevices of massive living coral colonies (*Porites* spp. and *Heliopora* spp. in particular).

**Figure 3:**
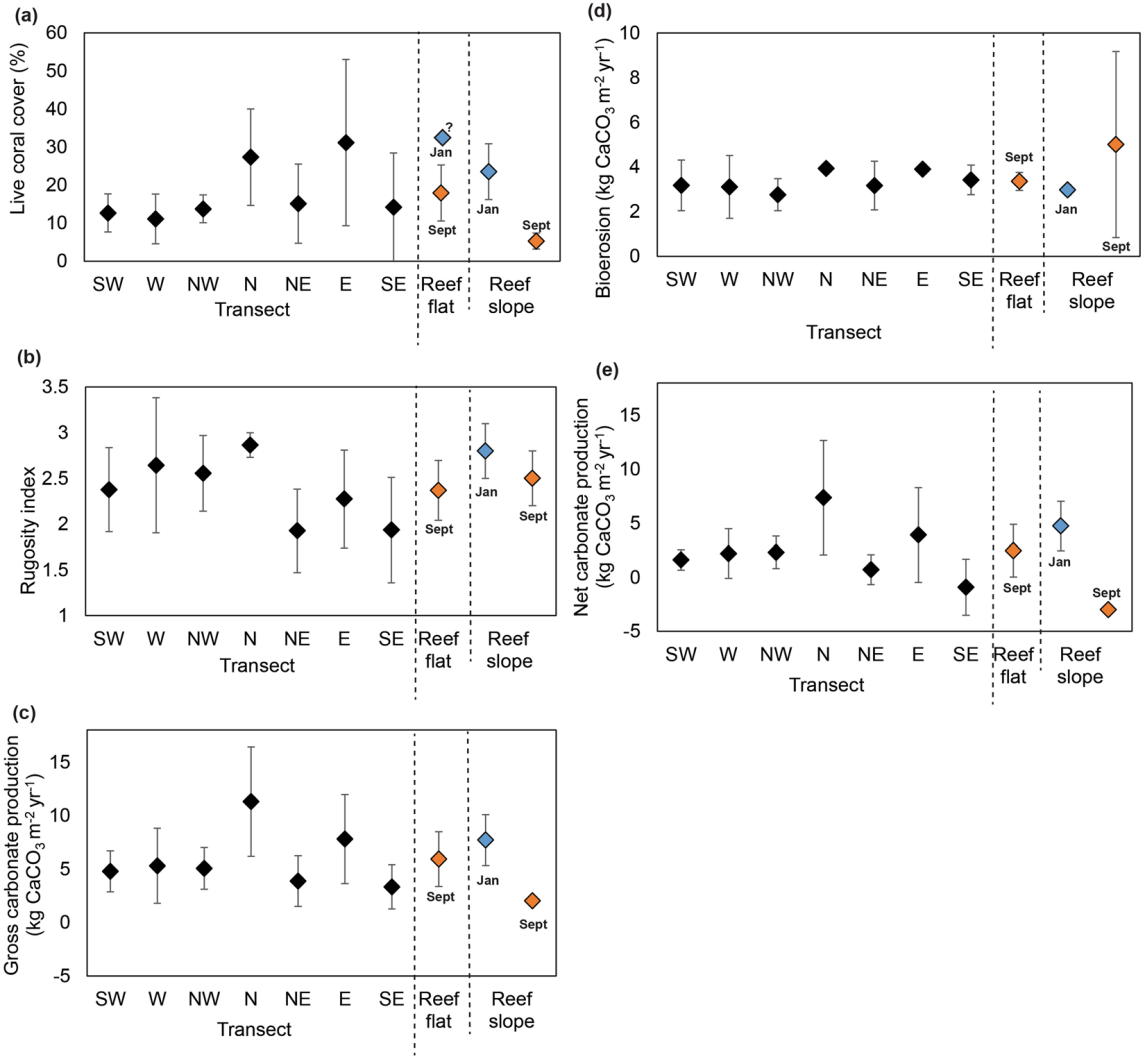
Average live coral cover (**a**), reef flat rugosity index (**b**), gross carbonate production (**c**), total bioerosion (**d**), and net carbonate production (**e**) across Mahutigala reef flat. Error bars are one standard deviation. Average reef flat values are indicated alongside average fore-reef slope (2 m depth) values measured in January 2016 (blue) and September 2016 (orange) by Perry and Morgan^16^. Reef flat live coral cover data is estimated for January 2016 (indicated by ‘?’) based on unpublished data that was collected in February 2016 by postgraduate students from the University of Auckland.

**Figure 4:**
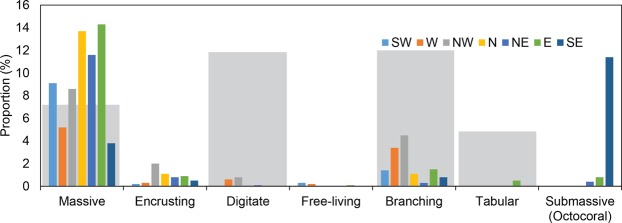
Coral growth morphologies as a proportion of the total average live coral cover recorded on the Mahutigala reef flat. Each transect (labelled) is shown by a different colour. Light grey shaded boxes represent estimated pre-bleaching average live coral cover recorded on the Mahutigala reef flat in February 2016 (unpublished data collected in February 2016 by postgraduate students from the University of Auckland).

Results of surveys conducted in September and October 2016 revealed that the live coral cover averaged for the entire Mahutigala reef flat was 17.9 ± 7.3% (mean, 1 standard deviation), ranging from 11.1 ± 6.5% at the W transect to 31.2 ± 21.8% at the E transect (Figs 2 and 3a). Typically, coral cover was highest in the northern to south-eastern sectors of the reef platform, where average live coral cover was 40.0%, 28.7% and 53.0% in the patch coral zone (zone 2) of the N, NE and E transects respectively, and 37.3% on the inner reef flat (zone 3) of the SE transect (Fig. 2). Massive corals (predominantly *Porites* spp., but also *Goniopora* spp., *Platygyra* spp., *Goniastrea* spp. and *Favia* spp.) were the dominant contributor to live coral cover, comprising 66.3% of all live coral recorded on the reef flat (Fig. 4). Branching corals (including *Acropora* spp. and *Pocillopora sp*.) and the Octocoral *Heliopora coerulea* comprised 13.0% and 12.6% of all reef flat live coral, respectively, with the remaining 8.1% comprised of small colonies of encrusting, digitate, free-living and tabular corals of varying genera (including *Fungia* spp., *Montipora* spp., *Porites* spp., *Galaxea* spp., among other unidentified genera) (Fig. 4).*H*. *coerulea* was most abundant on the SE transect, comprising 11.4% of all recorded live coral on the reef flat.

Reef substrate rugosity was calculated as a measure of the three-dimensional structural complexity of the reef ^36^. The average reef rugosity coefficient across the carbonate-producing zones of Mahutigala reef flat (zones 2–5) was 2.6, with averages ranging from 1.9 at the NE and SE transects and 2.9 at the N transect (Fig. 3b). Structural complexity was highest on the south-west to northern portion of the reef platform (Fig. 3b). Considering the measured reef rugosity coefficient and specific coral calcification rates for particular coral growth morphologies (Supplementary Materials), gross carbonate production was estimated to average 5.9 ± 2.5 G on Mahutigala reef platform. The most productive areas of the reef surface were in the north, where gross carbonate production was calculated as 11.3 ± 5.1 G and east (7.8 ± 4.2 G). The least productive area was the southeastern sector of the platform, where gross carbonate production was 3.3 ± 2.1 G (Supplementary Materials).

### Net carbonate budget

The total averaged biological erosion rate on the Mahutigala reef flat was estimated as 3.3 ± 0.4 G, with rates varying from 2.8 ± 0.7 to 3.9 ± 0.2 G across different radial transects (Fig. 3d). Parrotfish were the dominant contributors to biological erosion, contributing on average 94.1% of the total bioerosion rate (Supplementary Materials). Together, micro- and macro-borer erosion was estimated at 5.8% of the total bioerosion. Urchins (*Diadema antillarum* and *Phyllocanthus imperialis*) were uncommon across the reef platform (0.1 individual/m^2^ recorded on average). All observed *D*. *antillarum* were within the 21 to 40 mm test size range, while *P*. *imperialis* were all within the 81 to 100 mm test size range. On average, echinoderms contributed only 0.1% to bioerosion.

**Figure 5:**
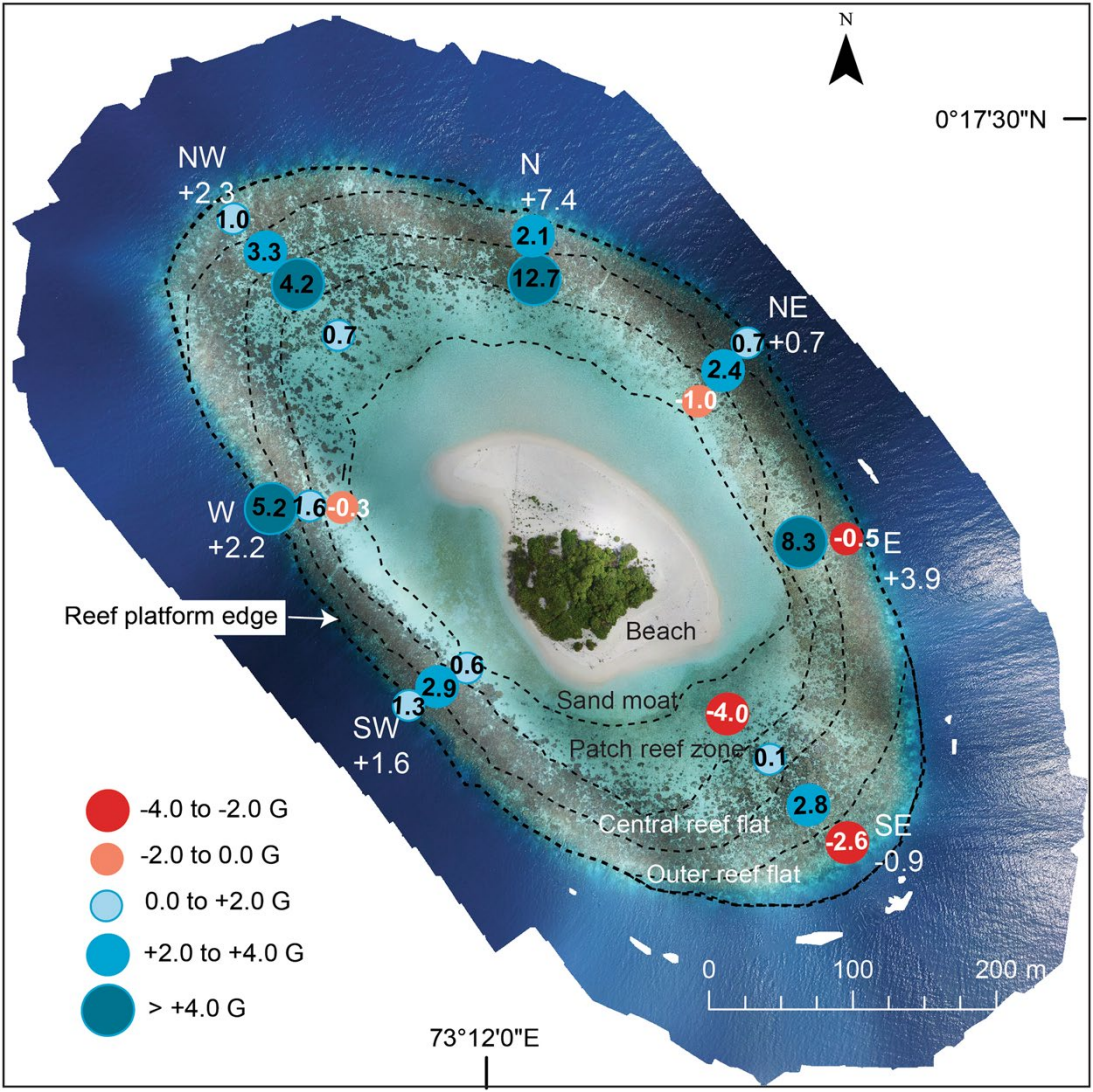
Average net carbonate budget rates (G) in different eco-geomorphologic zones around Mahutigala reef flat shown by coloured circled. Red-shaded circles represent areas of net negative carbonate budget, while blue-shaded circles represent areas of net positive carbonate budget. The average net carbonate budget for each radial transect (labelled) is shown in white text at the seaward end of the transect.

Net carbonate production thus averaged 2.5 ± 2.4 G across the Mahutigala reef platform surface. While the carbonate production state was positive, on average reef erosion and accretion were balanced, given the considerable spatial variation in net G values on the platform, ranging from a maximum of 7.3 ± 5.3 G in the north to a minimum of −0.9 ± 2.3 G (negative budget) at the SE transect (Fig. 5).

## Discussion

Our results present new insights of the effects of bleaching on the carbonate budget of a reef platform surface, expanding the limited number of budget studies in upper reef flat environments. Coral reefs experienced the third mass coral bleaching event in 2015–2016, causing significant declines in live coral cover, reef productivity and reef carbonate budgets in different regions^10,14^, including in the Maldives, Indian Ocean^13,16^. Existing reports of the impacts of this event are focused solely from observations of the shallow fore-reef (2–5 m depth). Upper reef platform surfaces (reef crest and reef flat) are often neglected in studies that explore reef carbonate budgets, despite the upper reef surface acting as the carbonate factory for islands, and arguably being more important than the fore-reef slope in acting as a dissipative barrier to incoming wave energy^1^ and thus exerting strong control on the wave energy that reaches adjacent shorelines. Thus, the effects of the 2015–2016 bleaching event on reef crest and reef flat carbonate production are essential to consider, particularly in light of recent suggestions that declines in reef productivity and carbonate budgets could lead to a reduction in reef accretion potential. This could exacerbate reef island wave overtopping and associated erosion due to enhanced wave access to shorelines and sea-level rise^16^ and also affect the sediment supply to island shorelines.

Results of ecological surveys conducted four months after the 2016 bleaching event in the southern Maldives showed the Mahutigala reef platform surface had an average live coral cover of 17.9 ± 7.3%. Erosion and accretion were essentially balanced at Mahutigala reef platform, where an average net positive carbonate budget of 2.5 ± 2.4 G was recorded (Fig. 3). Notably, the net carbonate budget was positive at most areas of the reef platform surveyed (Fig. 5), despite major collapse in the contribution of digitate (97% reduction), branching (75% reduction) and tabular corals (98% reduction) to the overall average reef surface live coral cover. The collapse of coral colonies occurred between January and September 2016 (Fig. 4) and was driven by bleaching associated coral mortality, as a direct consequence of the high SST anomaly event affecting the reef between March and May 2016^16^. This inferred coral collapse is supported by field observations prior to the bleaching event and a recent study of several fore-reefs on lagoonal platforms in Huvadhoo Atoll (including the Mahutigala reef slope) that records a significant decline of 91% in branching and tabular *Acropora* spp. corals after the high SST anomaly event^16^. Despite high mortality of branching corals on the reef surface, observations indicate that the structural complexity of the reef platform surface remained comparable to pre-bleaching levels (Fig. 3b). However, reef structural complexity is likely to have declined since September 2016 and is expected to continue to decline over time due to a combination of physical and biological erosion of dead coral. Indeed, rugosity reductions have been recorded on the SW fore-reef slope at Mahutigala^13^, whereby rugosity underwent a statistically significant decline between January 2016 (rugosity index of 2.7) and March 2017 (rugosity index of 2.0). However, expected reductions in structural complexity on the reef platform surface may be of a reduced magnitude than declines recorded on the reef slope, due to the predominance of massive corals on the reef flat, with lower surface-area ratios compared to branching and digitate corals, which dominated the fore-reef environment prior to the bleaching event^16^.

The positive post-bleaching net carbonate budget (average of all radial transects) recorded for Mahutigala reef platform surface of 2.5 ± 2.4 G, is up to 180% greater than the negative post-bleaching carbonate budget recorded for the Mahutigala reef slope (2 m depth contour, −3.0 ± 0.4 G, Fig. 3) by Perry and Morgan^16^, who used a comparable line transect methodology. The high spatial coverage of the line intercept surveys (6,300 observations) used in this study across all orientations of the reef provides confidence to conclude that the majority of the Mahutigala upper reef platform is characterized by a positive carbonate budget. In contrast, Perry and Morgan’s^16^ survey was focused on only one sector of Mahutigala (the southwest) and interestingly, their recorded net negative G values are in accord with the nearby SE transect of the reef surface in this study, where net negative G (−0.9 ± 2.3 G) was also observed (Fig. 5). This was notably the lowest transect-averaged net G value of the entire reef flat. Whether Perry and Morgan’s^16^ negative carbonate budget reported for the southwest orientation of the reef slope is representative of the entire Mahutigala fore-reef slope or not remains unknown. To resolve this question, additional ecological census surveys on all sectors of the fore-reef slope would be required to determine net carbonate production.

Net G values recorded on the southeastern and southwestern portions of the reef platform surface (−0.9 ± 2.3 G and 1.6 ± 0.9 G, respectively) were notably higher than that of the southwestern fore-reef slope^16^ (−3.0 ± 0.4 G). The higher net G values on the reef upper surface are directly attributable to the higher live coral cover (up to 37.3% and 19.7%) at the southeastern and southwestern reef platform, respectively (Fig. 2), compared with up to 7.0% at the southwestern fore-reef slope^16^. Furthermore, we argue that the relative dominance of massive corals (largely *Porites spp*., *Goniopora spp*. and the Octocoral *H*. *coerulea*) to the overall reef platform surface live coral cover is crucial to maintaining a positive carbonate budget following the bleaching event. The massive corals displayed strong resilience to the high SST anomaly event and did not experience mass mortality due to coral bleaching. However, it remains unclear whether these massive colonies temporarily bleached and recovered, or did not bleach at all.

The northern and eastern sectors of the reef platform displayed the highest average net G values (7.3 ± 5.3 G and 3.9 ± 4.9 G at transects N and E, respectively, Fig. 5) and also contained the highest proportion of massive live coral cover (up to 14% of the entire reef flat live coral cover; Fig. 4). This finding may be related to exposure and the hydrodynamic regime around the reef island, promoting massive coral dominance in the northern and eastern sectors. Mahutigala reef is likely influenced by a combination of lagoonal wind-driven wave energy and residual ocean swell energy. The southeastern and north-northeastern sectors of the reef likely receive the greatest wave energy. Ocean swell typically propagates towards Huvadhoo Atoll from the south or southeast^46^ and can reach the southeastern sector of Mahutigala reef by penetrating through the reef rim passage located 11 km to the southeast of Mahutigala. The northern sector of Mahutigala has a 50 m lagoonal fetch that likely generates significant internal wind-driven waves that impact the reef. Consequently, we infer exposure and hydrodynamic setting as a potential reason for the discrepancy in net G and massive coral dominance around Mahutigala. This highlights an important research gap that could be the focus of future investigations.

The differential survival of coral genera on the reef platform surface, with massive corals persisting and high mortality of branching, digitate and tabular corals, has led to marked changes in the reef community composition and structure, comparable to observations in the Maldives^25,47,48^, Japan^49^ and French Polynesia^50^ after the 1998 global coral bleaching event. Branching corals are typically more susceptible to bleaching than massive corals^11,51^ (though this is not always the case; see^52^) and several hypotheses have been proposed for explaining this preferential resilience including: greater tissue thickness of massive corals providing protection for the symbiotic alga within the coral tissue; and greater opportunity for mass transfer of toxins that accumulate during bleaching in massive, flat colonies compared to branching colonies^49,51^. Despite the shift in reef platform coral composition that has occurred at Mahutigala, from branching/digitate dominance to massive dominance, the platform surface still boasts a positive net carbonate budget, which has important implications for considering reef accretion potential.

**Figure 6:**
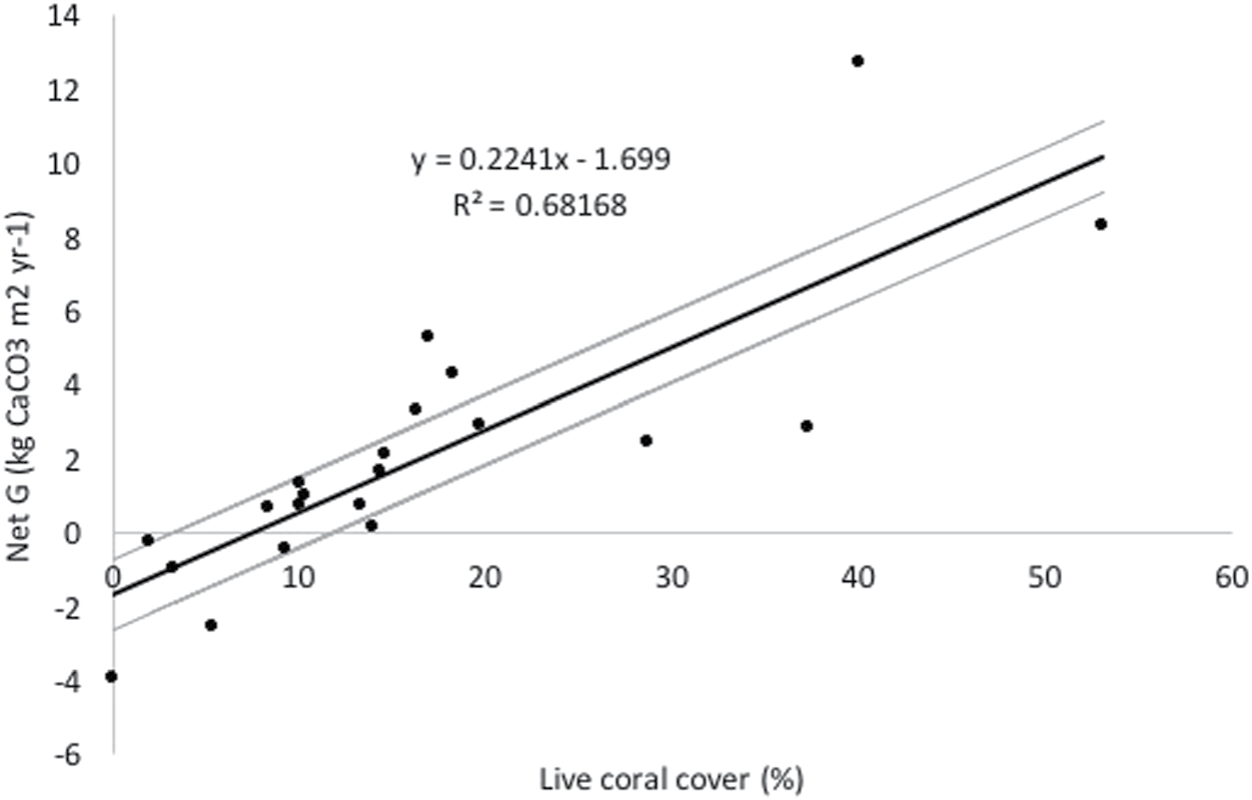
Relationship between live coral cover and net carbonate production (G) at Mahutigala. The linear regression is shown by the black line, with the 95% confidence interval (grey lines).

Carbonate budgets have been increasingly used to approximate reef accretion potential^12,16^ with recent studies suggesting that many coral reefs do not currently display vertical accretion rates sufficient to keep pace with projected rates of global sea-level rise^12^. However, the contribution of reef platform surface accretion is not considered in such studies, despite the reef flat acting as the critical geomorphic sub-component of a reef for wave energy dissipation^1,40^. Perry and Morgan^16^ identify a live coral cover threshold of around 12.0% for maintaining a net positive carbonate budget and a live coral cover threshold of around 8.0% for maintaining net positive reef accretion potential. Their threshold value for maintaining a net positive carbonate budget (12.0%) is comparable to that based on our data from Mahutigala reef flat of around 10.0% (Fig. 6). Thus, the threshold of 8.0% live coral cover for maintenance of net positive reef accretion potential can be considered suitable for the reef platform surface. Using this threshold value, the majority of Mahutigala reef surface is expected to maintain net positive accretion potential, despite high live coral mortality attributed to the 2016 bleaching event (Fig. 2). Provided a net positive accretion potential is maintained, the reef surface should continue to provide an effective buffer to incoming wave energy reaching the reef island shoreline and sea-level rise, contrary to suggestions based on data from Mahutigala fore-reef slope^16^. However, this will be highly dependent on many interacting and poorly understood factors and/or uncertainties that are beyond the aims of this study and require further research, including: future relative rates of reef accretion and sea-level rise; rates of carbonate sediment production, supply and incorporation into or removal from the reef structure in different geomorphic environments (e.g. fore-reef slope, upper reef flat, sand moat and lagoonal flats); the morphologic response of the reef island to future sea-level rise and associated changes in hydrodynamic regimes; and the impacts of various anthropogenic activities on these natural processes.

Our results show that carbonate production and thus reef accretion potential of the reef platform surface is different to the shallow fore-reef slope. Recognition of differential carbonate budget states between the fore-reef and platform surface has implications for future development of the entire reef platform and how carbonate budget studies should be interpreted. Firstly, differential accretion potential may lead to increased steepening of the platform structure. Secondly, caution must be exercised in extrapolating budget studies from discrete fore-reef zones to entire reef platform structures where spatial differences in coral cover and coral growth morphology exist. Finally, recovery of the branching and digitate corals will govern the timeframe in which spatial disparities in carbonate budget states persist. Reef ecosystem functionality will also be influenced by recovery of branching and digitate corals, as they are rapid regenerators, act as reef rugosity enhancers and provide shelter for reef organisms^53^, ultimately increasing reef biodiversity. At Mahutigala branching and digitate corals together comprised 14.5% of the total reef flat live coral composition in September 2016 (Fig. 4). Visual observations confirmed that the majority of branching and digitate live corals on the reef surface were juveniles and this indicates that the ability for recruitment and regeneration of branching and digitate corals did exist in September 2016. Further visual observations of Mahutigala reef flat by authors E.R. and P.K. in February 2017 and 2019 suggest that branching coral recruit abundance had increased since September 2016, indicating that the case for reef recovery may be positive. However, further data are needed and reef recovery will also depend on the timeframe of recurring stress events, including future coral bleaching, with respect to the timeframe of recovery. We anticipate recovery could take up to a decade based on coral recovery timeframes post the 1998 coral bleaching event in the central Maldives archipelago^25^ and assuming no major stress events during that timeframe. Moreover, if predictions of future increases in the return-period of mass coral bleaching events^10,54^ become reality, the recovery of branching and digitate corals at Mahutigala would be significantly hampered, and may result in fundamental ecological phase shifts. Without recovery of these corals, reefs may transform into a persistent massive coral dominated state. The impact of such a phase shift on the future reef carbonate budget and accretionary potential is unknown, although this study has shown that a positive biological carbonate budget can be maintained despite the loss of branching and digitate corals.

## Methods

### Gross carbonate budget

Ecological surveys were conducted at Mahutigala reef (0°17′22.77′′N, 73°12′1.88′′E, Fig. 1), Huvadhoo Atoll, southern Maldives, in September and October 2016, following the south-west monsoon. The Mahutigala reef platform is approximately 0.13 km^2^ in area, containing a 0.004 km^2^ vegetated island (elevated 1.5–2.0 m above mean sea level), surrounded by an island beach, lagoon and elliptical shaped reef platform. Reef flat eco-geomorphological zones were visually differentiated across seven shore-perpendicular radial transect lines (SW, W, NW, N, NE, E, SE, Fig. 1), according to variations in geomorphology, substrate and live coral cover. To quantify reef flat benthic composition, ecological surveys were undertaken within each eco-geomorphic zone along the seven radial transects using a modified version of the ReefBudget^18^ methodology. Three replicate 10 m survey lines were randomly established using a flexible tape measure fixed to the substrate in each of the eco-geomorphic zones across each radial transect line. A total of 63 10 m lines were surveyed (equating to 6300 observations). To characterize benthic cover, observations were made at 10 cm intervals along the 10 m survey lines (100 observations per survey line) using the Point Intercept Transect method^33,55^, whereby the nature of the benthic cover directly below the tape measure at each 10 cm increment was recorded (e.g. substrate, primary/secondary carbonate producers, algae, reef framework, dead coral). Scleractinian corals were recorded to growth morphology (massive, branching, tabular, digitate, encrusting and free-living) and genus level where feasible. Pre-bleaching average coral cover (%) is estimated based on unpublished data collected by postgraduate students from the University of Auckland in February 2016. Coral cover data were collected using the same Point Intercept Transect method, where observations of substrate cover were made at 10 cm intervals. Substrate rugosity was quantified along three randomly selected 1 m sections of each 10 m survey line using the chaining technique, whereby the total substrate surface length is divided by 1 m linear length of chain^36^. The three rugosity values were averaged to determine a rugosity coefficient (R) for each 10 m survey line. Gross carbonate production rates (G, or kg CaCO_3_ m^−2^ yr^−1^) for corals and coralline algae were estimated for each 10 m survey line using genera and coral morphology specific skeletal density and linear growth rates (Supplementary Materials) using the following equation after^33^ and^18^:

1 $${\boldsymbol{G}}=(R\times ((Xi/100))\times ((Ci\times 10,000)/1000)))$$

where *X*_*i*_ is the mean percent cover of the *ith* genera. The calcification rate (*C*_*i*_) of the *ith* genera was estimated in g cm^−2^ y^−1^ from mean published coral and coralline algae *C*_*i*_ rates from the Maldives (with two exceptions from the Indo-Pacific; see Supplementary Materials).

### Net carbonate budget

Biological erosion rates were conservatively estimated using a combination of published data and *in situ* surveys. To quantify macro- and micro-borer erosion rates (G, kg m^−2^ yr^−1^) by endolithic organisms, regional Indo-Pacific published rates were used (0.052 G for macro-borer erosion and 0.053 G for micro-borer erosion after^33,56^ and applied to all available reef substrate (live coral, reef framework, dead coral and rubble) using equation:

2 $$Bioerosion\,rat{e}_{borer}(G)=R\times  \% available\,substrate\times 0.053$$

To calculate bioerosion by parrotfish (G) we used published rates^16^ based on belt transect surveys conducted at the Mahutigala fore-reef slope (2 m depth) in September 2016. Perry and Morgan^16^ estimated parrotfish bioerosion at Mahutigala as 3.81 ± 4.17 G and this rate was applied to all available substrate at Mahutigala reef flat. Uncertainties must be considered around using this published parrotfish erosion rate for the fore-reef slope and applying it to available substrate at Mahutigala reef flat. These uncertainties include disparities in parrotfish abundance, species and size class on the fore-reef slope compared with the reef flat, associated variability in bioerosion rates by fish of different species and size classes^57^, and unknown spatial differences in parrotfish abundance or potential feeding location preferences on the Mahutigala reef flat.

Echinoid bioerosion rates were quantified within each of the productive eco-geomorphic zones on transects SW, NW, NE and SE at Mahutigala reef flat along two replicate 30 m-long by 2 m-wide belt transects. Urchin abundance and genera were recorded along the belt transects. Echinoid bioerosion rates (G) for *Diadema* spp. and *Phyllocanthus* spp. were calculated as a function of urchin genera and size, following^18^ using the equations:

3 $$Bioerosion\,rat{e}_{Diadema}(G)=\frac{(\frac{((0.0029\times {X}^{1.6624})\times e)\times 365}{60})}{1000}$$

4 $$Bioerosion\,rat{e}_{Phyllocanthus}\,(G)=\frac{(\frac{((8\times {10}^{5}\times {X}^{1.6624})\times e)\times 365}{60})}{1000}$$

where *X* is the average echinoid test size (mm) and *e* is the echinoid density in the 30 m^2^ belt transect. The average echinoid test size (mm) was 30 mm and 90 mm for *Diadema* spp. and *Phyllocanthus spp*., respectively. Macro- and micro-borer, parrotfish, and echinoid bioerosion rates were all extrapolated to the available reef substrate for erosion at Mahutigala and summed to provide an estimate rate (G) for total annual bioerosion. Net carbonate production rates (G) were determined by subtracting total bioerosion from gross production.

## Supplementary information

Supplementary Information
