# Supplementary material for: Massive corals maintain a positive carbonate budget of a Maldivian upper reef platform despite major bleaching event
Source: Sci Rep. 2019 Apr 24;9:6515. doi: 10.1038/s41598-019-42985-2 (PMC6482145; doi:10.1038/s41598-019-42985-2)
Supplement: Supplementary file 1 — Supplementary Information [file 41598_2019_42985_MOESM1_ESM.docx]

**Massive corals maintain a positive carbonate budget of a Maldivian upper reef platform despite major bleaching event**

E.J. Ryan^1*^, K. Hanmer^1^, and P.S. Kench^1,2^

^1^ School of Environment, the University of Auckland, Auckland, 1010, New Zealand

^2^ Department of Earth Sciences, Simon Fraser University, BC, Canada
* Corresponding Author

**Supplementary Materials**

This document contains tables and figures presenting supporting data and information.

**Table 1** Summary data on Mahutigala reef platform surface carbonate production for different radial transects and the entire reef platform. Averages and one standard deviation presented. G = kg CaCO_3_ m^2^ yr^-1^.

| Transect | **SW** | **W** | **NW** | **N** | **NE** | **E** | **SE** | **Reef platform average** |
| --- | --- | --- | --- | --- | --- | --- | --- | --- |
| Rugosity | 2.4 ± 0.5 | 2.6 ± 0.7 | 2.6 ± 0.4 | 2.9 ± 0.1 | 1.9 ± 0.5 | 2.3 ± 0.5 | 1.9 ± 0.6 | 2.4 ± 0.3 |
| Live coral cover (%) | 12.7 ± 5.0 | 11.11 ± 6.5 | 13.7 ± 3.4 | 27.3 ± 12.7 | 15.11 ± 10.4 | 31.2 ± 21.8 | 14.2 ± 14.3 | 17.9 ± 7.3 |
| Gross coral production (G) | 4.0 ± 1.7 | 5.0 ± 3.2 | 4.8 ± 2.0 | 10.8 ± 5.3 | 3.7 ± 2.3 | 7.6 ± 4.3 | 2.4 ± 2.3 | 5.5 ± 2.6 |
| Gross CCA production (G) | 0.3 ± 0.2 | 0.1 ± 0.1 | 0.1 ± 0.0 | 0.2 ± 0.1 | 0.0 ± 0.0 | 0.1 ± 0.1 | 0.1 ± 0.0 | 0.1 ± 0.1 |
| Gross *Halimeda* production (G) | 0.5 ± 0.5 | 0.3 ± 0.2 | 0.2 ± 0.1 | 0.3 ± 0.1 | 0.2 ± 0.2 | 0.1 ± 0.1 | 0.1 ± 0.0 | 0.2 ± 0.2 |
| Gross carbonate production (G) | 4.8 ± 1.9 | 5.3 ± 3.5 | 5.1 ± 2.0 | 11.3 ± 5.1 | 3.9 ± 2.4 | 7.8 ± 4.2 | 3.3 ± 2.1 | 5.9 ± 2.6 |
| Parrot fish bioerosion (G) | 2.9 ± 1.0 | 2.9 ± 1.3 | 2.6 ± 0.6 | 3.6 ± 0.2 | 3.0 ± 1.0 | 3.7 ± 0.2 | 3.2 ± 0.6 | 3.1 ± 0.4 |
| Micro and macro borer bioerosion (G) | 0.2 ± 0.1 | 0.2 ± 0.1 | 0.2 ± 0.1 | 0.3 ± 0.0 | 0.2 ± 0.1 | 0.2 ± 0.1 | 0.2 ± 0.1 | 0.2 ± 0.0 |
| Echinoderm bioerosion (G) | 0.0 ± 0.0 | 0.0 ± 0.0 | 0.0 ± 0.0 | 0.0 ± 0.0 | 0.0 ± 0.0 | 0.0 ± 0.0 | 0.0 ± 0.0 | 0.0 ± 0.0 |
| Total bioerosion (G) | 3.2 ± 1.1 | 3.1 ± 1.4 | 2.8 ± 0.7 | 3.9 ± 0.2 | 3.2 ± 1.1 | 3.9 ± 0.2 | 3.4 ± 0.7 | 3.3 ± 0.4 |
| Net carbonate production (G) | 1.6 ± 0.9 | 2.2 ± 2.3 | 2.3 ± 1.5 | 7.3 ± 5.3 | 0.7 ± 1.4 | 3.9 ± 4.9 | -0.9 ± 2.3 | 2.5 ± 2.4 |

**Table 2** Summary of published calcification rates used in this study to calculate gross carbonate production on Mahutigala reef platform surface.

|  | Genus/Species or growth morphology | Average calcification rate (g cm^-2^ y^-1^) (1 sd) | Location | Reference |
| --- | --- | --- | --- | --- |
| Scleractinian coral | Massive | 1.38 ± 0.33 | Vabbinfaru, Male Atoll, Maldives | Morgan and Kench (2012) |
|  | Encrusting | 0.31 ± 0.13 | Vabbinfaru, Male Atoll, Maldives | Morgan and Kench (2012) |
|  | Digitate | 1.61 ± 0.85 | Vabbinfaru, Male Atoll, Maldives | Morgan and Kench (2012) |
|  | Free-living | 1.75 ± 0.63 | Vabbinfaru, Male Atoll, Maldives | Morgan and Kench (2012) |
|  | Branching | 1.75 ± 0.63 | Vabbinfaru, Male Atoll, Maldives | Morgan and Kench (2012) |
|  | Tabular | 1.92 ± 0.85 | Vabbinfaru, Male Atoll, Maldives | Morgan and Kench (2012) |
| Octocoral | *Heliopora coerulea* | 0.48 ± 0.00 | Shiraho Reef, Ishigaki, Japan | Planck et al. (1988) |
| Coralline algae | *Halimeda* spp. | 0.18 ± 0.07 | Great Barrier Reef, Australia | Drew (1983); Drew and Abel (1988) |
|  | Crustose coralline algae | 0.05 ± 0.02 | Vabbinfaru, Male Atoll, Maldives | Morgan and Kench (2012) |

**List of references**

Drew, E.A. Halimeda biomass, growth rates and sediment generation on reefs in the central Great Barrier Reef province. *Coral Reefs.* **2**,101–110 (1983).

Drew, E.A. & Abel, K.M. Studies on Halimeda 2. Reproduction, particularly the seasonality of gametangia formation, in a number of species from the Great Barrier Reef Province. *Coral Reefs.* **6**, 207–218 (1988).

Morgan, K.M. & Kench, P.S. Skeletal extension and calcification of reef-building corals in the central Indian Ocean. *Mar Env Res.* **81**, 78–82 (2012).

Planck, R.J., McAllister, D.E. & McAllister, A.T. Shiraho coral reef and the proposed new Ishigaki Island Airport, Japan. *International Union for Conservation of Nature and Natural Resources,* Morgas Switzerland.
